# Information Presentation Features and Comprehensibility of Hospital Report Cards: Design Analysis and Online Survey Among Users

**DOI:** 10.2196/jmir.3414

**Published:** 2015-03-16

**Authors:** Uwe Sander, Martin Emmert, Jochen Dickel, Nina Meszmer, Benjamin Kolb

**Affiliations:** ^1^Department of Information and CommunicationFaculty for Media, Information and DesignUniversity of Applied Sciences and Arts HannoverHannoverGermany; ^2^Institute of Management (IFM)School of Business and EconomicsFriedrich-Alexander-University Erlangen-NurembergNurembergGermany; ^3^Faculty of MediaUniversity of Applied Sciences FHMBielefeldGermany

**Keywords:** public reporting, report cards, information presentation

## Abstract

**Background:**

Improving the transparency of information about the quality of health care providers is one way to improve health care quality. It is assumed that Internet information steers patients toward better-performing health care providers and will motivate providers to improve quality. However, the effect of public reporting on hospital quality is still small. One of the reasons is that users find it difficult to understand the formats in which information is presented.

**Objective:**

We analyzed the presentation of risk-adjusted mortality rate (RAMR) for coronary angiography in the 10 most commonly used German public report cards to analyze the impact of information presentation features on their comprehensibility. We wanted to determine which information presentation features were utilized, were preferred by users, led to better comprehension, and had similar effects to those reported in evidence-based recommendations described in the literature.

**Methods:**

The study consisted of 5 steps: (1) identification of best-practice evidence about the presentation of information on hospital report cards; (2) selection of a single risk-adjusted quality indicator; (3) selection of a sample of designs adopted by German public report cards; (4) identification of the information presentation elements used in public reporting initiatives in Germany; and (5) an online panel completed an online questionnaire that was conducted to determine if respondents were able to identify the hospital with the lowest RAMR and if respondents’ hospital choices were associated with particular information design elements.

**Results:**

Evidence-based recommendations were made relating to the following information presentation features relevant to report cards: evaluative table with symbols, tables without symbols, bar charts, bar charts without symbols, bar charts with symbols, symbols, evaluative word labels, highlighting, order of providers, high values to indicate good performance, explicit statements of whether high or low values indicate good performance, and incomplete data (“N/A” as a value). When investigating the RAMR in a sample of 10 hospitals’ report cards, 7 of these information presentation features were identified. Of these, 5 information presentation features improved comprehensibility in a manner reported previously in literature.

**Conclusions:**

To our knowledge, this is the first study to systematically analyze the most commonly used public reporting card designs used in Germany. Best-practice evidence identified in international literature was in agreement with 5 findings about German report card designs: (1) avoid tables without symbols, (2) include bar charts with symbols, (3) state explicitly whether high or low values indicate good performance or provide a “good quality” range, (4) avoid incomplete data (N/A given as a value), and (5) rank hospitals by performance. However, these findings are preliminary and should be subject of further evaluation. The implementation of 4 of these recommendations should not present insurmountable obstacles. However, ranking hospitals by performance may present substantial difficulties.

## Introduction

### Background

In recent years, many health care systems have implemented strategies to improve the quality of care [1]; nevertheless, quality deficits and variability still remain [1-6]. In general, patients are unlikely to be aware of the existence of quality differences [7,8]. One reason for this is the limited amount of publicly available information on the quality of health care providers [9]. Therefore, improving the transparency of information about health care provider quality is a major challenge [10]. It is assumed that this will improve overall quality by steering patients toward better-performing health care providers [11,12] and by incentivizing providers to improve quality [8,9,13-15].

Public reporting in Germany is partly regulated by law. Since 2005, German hospitals have had to publish quality reports online to help patients and physicians make informed choices of hospitals. The AQUA Institute has been commissioned to make further improvements in quality assurance [16]. In 2011, 1666 hospitals had to participate in an “external quality assurance” process. Since the beginning of 2012, the quality reports of individual hospitals include 182 of 430 quality measures [16]. A possible quality shortfall at a hospital can trigger an evaluation, including a structured quality dialog, which allows a group of experts to conduct a qualitative investigation of discrepant results at individual hospitals. In 2010, a total of 21,053 discrepant results were identified in 4,064,320 datasets. Of these, 8.0% were evaluated as qualitatively discrepant through the structured quality dialog. The AQUA Institute does not report on individual hospitals, but German public reporting portals draw on the information provided in the quality reports of individual hospitals.

Nevertheless, there are several barriers to effective public reporting in Germany. Friedemann et al [17] analyzed quality reports of individual German hospitals and concluded that they were neither readable nor understandable for most patients. In international studies, 1 of the barriers most frequently discussed is that consumers do not understand the formats in which the information is presented. Hibbard [18] noted that we need to find more effective ways to present data to consumers. Similarly, Sinaiko et al [19] argued that the current report cards need to be substantially expanded and refined. Kullgren and Werner [20] added that the problem with current public reporting is partly caused by the limitations imposed by the design of report cards.

Although the number of public reporting websites is likely to continue to rise [21], many argue that in their current state they might confuse consumers. Rothberg and colleagues [22] even argued that it would be better to report nothing at all rather than misleading information. Similarly, Emmert et al [23] stated that patients or physicians should not yet use such information to choose an individual physician. In the rush to make providers accountable, enthusiasm has often outstripped science [22]. Several researchers have pointed to the tremendous diversity in the presentation of quality data [21]. The websites and related reports vary widely in terms of ease of access, ease of use, usefulness of information, timeliness of updates, and credibility [24].

### Research Questions

After reviewing the available international recommendations, we analyzed the 10 most commonly used German public report cards and addressed 3 questions:

1. What information presentation elements were utilized?
2. Which led to better comprehension?
3. Which had similar effects to those reported in the evidence-based recommendations described in literature?

We focused on elements of information design that are used to communicate ideas, illustrate information, or express relationships visually, such as pictures, symbols, and colors [25].

## Methods

### Overview

The study consisted of 5 steps: (1) identification of best-practice evidence about the presentation of information on hospital report cards, (2) selection of a single risk-adjusted quality indicator for the study, (3) selection of a sample of the public report card designs used by German hospitals, (4) identification of information presentation elements used in public reporting initiatives in Germany, and (5) conduct of an online-based survey.

### Identification of Best-Practice Evidence About the Presentation of Information on Hospital Report Cards

A literature search was conducted in April 2013 by searching the Medline (via PubMed) and Cochrane Library databases using the “Abstract/Title/Keywords” search field: Search (“Public Report$ OR Publicly Report$ OR Publicly Release$ OR Public Disclos$ OR Information Disseminat$ OR Report Card$ OR Consumer Report$ OR Quality Report$ OR Comparative Report$ OR Reporting Instrument$”); limitations: English, German, and Spanish; published in the last 10 years; field: “Title/Abstract/Keywords.” Only peer-reviewed journal articles and 3 types of review were included: (1) studies which compared health care report cards in terms of specific criteria, (2) studies which theoretically or empirically assessed or discussed the distinguishing features of public report cards and provided evidence on how performance data should be published or presented, and (3) studies which summarized or discussed best practice in public reporting for health care.

### Selection of a Single Risk-Adjusted Quality Indicator for the Study

Several risk-adjusted outcome quality measures were available from the German Hospital Quality Report 2011 [17]. The selection of the quality indicator for the study was done by assessing case numbers, the number of hospitals using the measure, and its role in quality assurance. We selected an elective procedure because publicly available information on elective treatments might be expected to help patients identify a good health care provider. We selected a risk-adjusted mortality rate (RAMR) measure because these are considered useful indicators of hospital quality [26-28].

### Selection of a Sample of the Public Report Card Designs Used by German Hospitals

To identify relevant hospital websites, we used a Google search by searching on several keywords (eg, *Kliniksuche* [clinic search], *Krankenhaussuche* [hospital search], *Gute Klinik* [good clinic], *Klinikvergleich* [compare clinic]). The most frequently visited hospital rating websites were identified using the Alexa analysis tool.

### Identification of Information Presentation Elements Used in Public Reporting Initiatives in Germany

The presentations of the selected quality indicators in 10 report cards were captured with screenshots that included interactive features (mouseovers). Information presentation elements were categorized with nVivo 10 by 2 authors using the previously identified literature-derived categories. Additional categories were added when no literature-derived categories were available.

### Conduct of an Online-Based Survey

We applied an online-based cross-sectional study by surveying an online panel to address the following questions:

1. Were respondents able to identify the hospital with the lowest RAMR?
2. Were respondents’ hospital choices associated with particular information design elements?

Consultation took place with an online panel in Germany in August-September 2013; each participant received €1 per finished survey. The panelists were recruited through several recruitment channels including online recruitment, direct mailing, and offline recruitment. The panel members were invited by email to participate (the invitation contained a link to the online survey). The survey was administered and conducted by Norstat Germany Ltd (formerly ODC Services Ltd), a fieldwork agency for survey research.

The online questionnaire consisted of 3 parts. First, a short introduction described the background and study objectives. Second, a random selection of 3 of the 10 hospital report cards was presented to prevent training effects (eg, overfamiliarization with presentation techniques leading to biased results). Respondents were asked to select the best quality hospital, to justify their choice in response to an open-ended question, and to assess the comprehensibility of the website (range of 1=very comprehensible to 7=very incomprehensible). Third, respondents provided sociodemographic data. The questionnaire was piloted and descriptive analyses were conducted using SPSS version 21.0 (IBM Corp, Armonk, NY, USA). The statistical significance of differences between responses was calculated using chi-square tests and *t* tests. Analysis of the open-ended questions was conducted by 2 authors coding and categorizing answers independently using nVivo 10; discrepancies were discussed to achieve a consensus.

## Results

### Identification of Best-Practice Evidence About the Presentation of Information on Hospital Report Cards

Our search yielded 2506 hits in the Cochrane Library and 1827 in PubMed. After elimination of duplicates, 4302 articles were screened by title and abstract, resulting in exclusion of 4018 articles. In addition, 7 studies were identified through reference search, expert consultation, and Internet searches, giving 291 articles for full-text review. Of these, 13 articles published between 2001 and 2013 met the inclusion criteria. Ten articles described observations in the United States, 2 in the Netherlands, and 1 in Germany.

Table 1 shows the literature-derived categories for the presentation of information on health care report cards. Because this study focused on outcome quality measures, only the information presented on these measures is described here. The methodological quality of the literature used is described in Multimedia Appendix 1.

**Table 1:** Features of the presentation of information on health care report cards from previous studies.

Category	Recommendations and results
Evaluative table with symbols	Consider using a table design such as the “evaluative table with stars” rather than a bar chart [29]
	Evaluative tables using words or stars are superior to numerical tables [29]
	Physicians preferred formats that used traffic light symbols to code the value of indicators (numerical table with traffic lights) [30]
Tables without symbols	Graphic displays were more helpful to users than text-only tables [31]
Bar charts	Bar charts were commonly used (43% of public reporting websites) [32]
Bar charts without symbols	Comprehension was lowest when data were presented in bar charts [30]
	Standard bar charts were not well-liked by respondents and led to the lowest levels of comprehension [29]
Bar charts with symbols	Symbols and bar charts should be used [31]
	A combination of bar charts and star ratings facilitated correct interpretation by users [32]
	Adding stars to bar charts increases comprehension significantly [33]
Symbols	Participants liked to use symbols to identify the best surgeon [31]
	Physicians preferred formats that used symbols (eg, traffic lights) [30]
	Star-only formats should be used in preference to numerical values [34]
	Only important information should be made easier to evaluate using symbols [35]
Evaluative word labels	Adding evaluative labels to bar charts did not increase comprehension [33]
Highlighting	Color-coding important information improves comprehension [36]
	Highlighting information about quality resulted in greater understanding [37]
	Presentation formats which highlighted key messages increased comprehension [38]
Order of providers	Physicians prefer presentation formats that combine individual indicator values with evaluative features such as rankings [30]
	Comprehension of respondents who were low in numeracy was significantly improved by the ordered compared to the unordered condition [35]
	Providers should be ranked by performance [12]
	Ranking plans by performance significantly decreased errors in interpreting data [33]
	Ranking by performance increased the frequency with which users chose higher-performing services [15]
	Providers should be ranked in descending order of quality, as this was valued by participants and increased their comprehension [36]
	One of the more powerful display strategies is to rank providers in terms of performance [33]
	When providers were ordered alphabetically participants were more likely to make effective use of the data (ie, choose the best provider) than when providers were ordered by performance [32]
High values indicate good performance	Performance data should be displayed such that high values always represent high performance [35]
	Numeric tables and bar charts often led respondents to conclude that the worst performing nursing homes (those with the higher percentages) were the best, notwithstanding the warning label at the top [29]
State explicitly whether high or low values indicate good performance	It should be stated explicitly whether high or low values indicate good performance, regardless of the direction of the scale [8,36]
Incomplete data (“N/A” as a value)	Incomplete data (missing values) have a negative influence on provider assessment and the potential to influence a decision [29]

### Selection of a Single Risk-Adjusted Quality Indicator for the Study

For our investigation, we selected the risk-adjusted quality indicator coronary angiography and percutaneous coronary intervention (PCI). This procedure was performed 715,469 times in 841 German hospitals in 2011. In 2011, 6369 of 276,866 (2.30%) patients died after PCI; 2.24% mortality had been expected, resulting in a RAMR of 1.03 [17].

### Selection of a Sample of the Public Report Card Designs Used by German Hospitals

A total of 63 hospital public reporting websites were identified by a Google search. Several report cards were eliminated because they did not present outcome quality measures or used presentation formats identical to those of sites already included in the sample. Of the remaining report cards, the 10 most frequently visited were used as a sample (Multimedia Appendix 2): Portal A [39], B [40], C [41], D [42], E [43], F [44], G [45], H [46], I [47], and K [48].

### Identification of Information Presentation Elements Used in Public Reporting Initiatives in Germany

**Figure 1:**
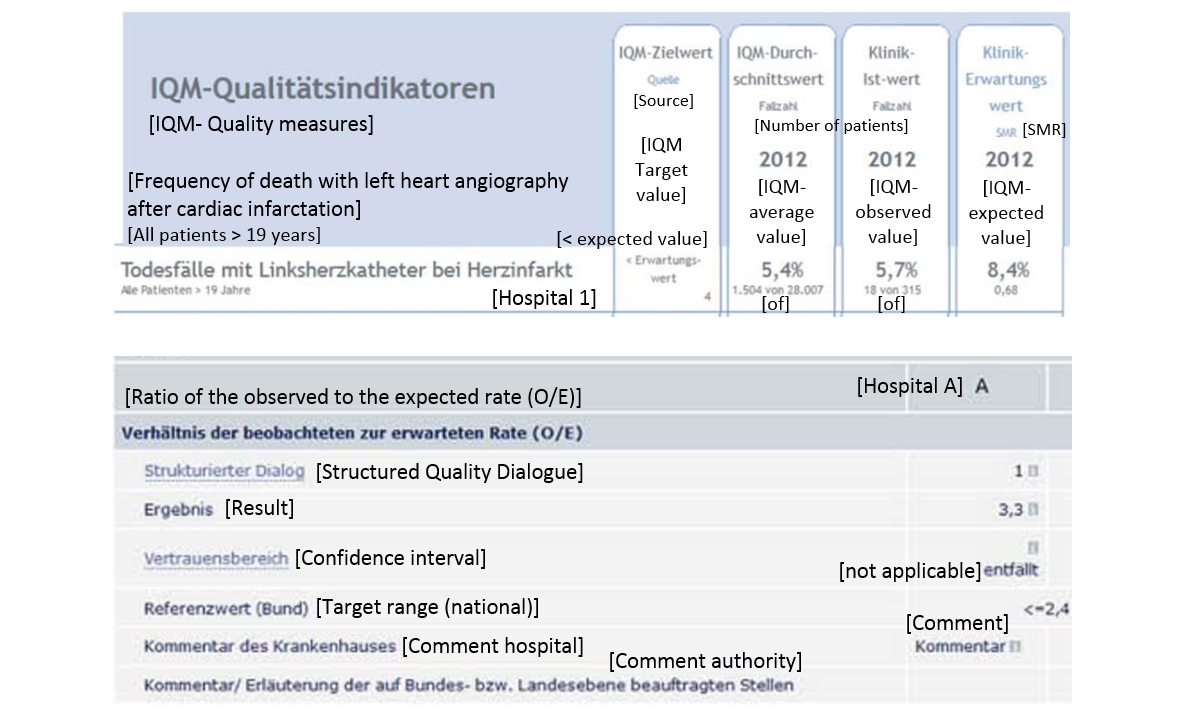
Tables without symbols. Top: Portal E; bottom: Portal G. Only results for hospital 1 are displayed. English translations in brackets.

The formats used to present information about RAMR in coronary catheterization by the 10 public reporting websites (see Multimedia Appendix 2) that we studied are summarized in Table 2. Tables (5 sites) and bar charts (5 sites) were equally popular; 4 sites presented reports with incomplete or missing data (“N/A” as a value). Symbols such as traffic lights were commonly used (7 sites), sometimes in combination with bar charts (4 sites) or tables (5 sites) (see Figure 1). Five report cards used low values (for the mortality rate) to indicate good performance and we identified 2 report cards which indicated a “good quality” range for the RAMR.

**Table 2:** Features used in the presentation of risk-adjusted mortality rates for coronary catheterization by 10 German portals.

Elements of information presentation	n	Portals
Table with symbols	3	B, I, K
Table without symbols	2	C, E,
Bar chart without symbols	1	G
Bar chart with symbols	4	A, D, F, H
Bar chart with traffic light symbols	3	A, D, H
Bar chart with thumb symbols	1	F
Symbols only	0	—
Evaluative word labels	0	—
Highlighting	0	—
Providers ranked by performance	2	D, H
High values indicating good performance	0	—
Explicit statement about whether high or low values indicate good performance	5	A, D, G, H, I
No statement about scale direction, but a “good quality” range identified	2	A, H
Incomplete data (“N/A” as a value)	4	B, C, F, K

### Conduct of an Online-Based Survey

In total, 3064 respondents started the online survey and 2027 completed it (completion rate=66.16%), taking a mean 14.51 (SD 9.39) minutes. The overall mean age of respondents was 41.57 (SD 15.87) years; 978 of 2027 respondents were female (48.24%) and 50.71% (1028/2027) graduated from high school or obtained a technical university entrance qualification (see Table 3). A total of 96.69% (1960/2027) used the Internet at least once a day. Table 3 also shows the results of the Arbeitsgemeinschaft Online Forschung (AGOF) Internet Facts 2014-07 survey of the German population who used the Internet in the past 3 months [49]. Comparing our survey results to those of the AGOF, the strongest difference appears to be in the demographics of the 2 surveys, specifically in the rate of persons without school qualifications or with secondary general school. In our survey, this rate was 11.69% (237/2027) compared to 35.2% in the AGOF survey. Differences in age, gender, and household size were weaker.

**Table 3:** Overview of the study sample in comparison with Internet users in Germany.^a^

Demographics			Study sample (N=2027)	Internet users in Germany (N=106,677)
**Age (years)**				
	Mean (SD)		41.57 (15.87)	—
	**Range, n (%)**			
		≤20	255 (12.58%)	(13.6%)
		21-30	334 (16.47%)	(17.1%)
		31-40	355 (17.51%)	(16.3%)
		41-50	484 (23.87%)	(20.6%)
		51-60	328 (16.18%)	(16.8%)
		≥61	270 (13.32%)	(15.5%)
Gender (female), n (%)			978 (48.24%)	(47.5%)
**Household size, n (%)**				
	1 person		456 (22.50%)	(16.8%)
	2 persons		725 (35.76)	(33.7%)
	3 persons and more		845 (41.69%)	(49.5%)
**Education, n (%)**				
	Still at school		67 (3.31%)	(4.7%)
	Without school qualification or secondary general school		237 (11.69%)	(35.2%)
	Intermediate secondary school or equivalent qualification		694 (34.24%)	(30.6%)
	High school graduation/technical university entrance qualification		1028 (50.72%)	(34.2%)

^a^ As measured by the Arbeitsgemeinschaft Online Forschung (AGOF) Internet Facts 2014-07 survey of the German population who used the Internet in the last past 3 months [49].

### Were Respondents Able to Identify the Hospital With the Lowest Risk-Adjusted Mortality Rate?

Table 4 shows the results of the survey. Three of the 10 report cards were presented to each respondent (N=2027) for a total of 6081 observations. For each site, a mean 60.68% (1230/2027) of respondents successfully identified the hospital with the lowest RAMR, and 6.81% (138/2027) (range 0.8%-14.0%) selected the hospital with the highest RAMR. In approximately 14.60% (296/2027) of all observations, respondents stated they were unable to provide an answer. Only 32.02% (649/2027) of respondents selected the hospital with the lowest RAMR on all 3 randomly assigned report cards, whereas 14.01% (284/2027) did not identify the hospital with the lowest RAMR on any report card.

**Table 4:** Respondents were asked to select the best quality hospital: overview of the selected hospitals (N=6081 observations).

Portal used	Which hospitals did the respondents select?, n (%)					
	Hospital 1	Hospital 2	Hospital 3	Hospital 4	Hospital 5	Could not answer
Portal A	123 (2.02)	4695 (77.21)^a^	282 (4.64)	208 (3.42)	195 (3.21)^b^	581 (9.55)
Portal B	224 (3.68)^b^	2238 (36.8)	480 (7.89)	144 (2.37)	1909 (31.39)^a^	1088 (17.89)
Portal C	766 (12.60)^b^	341 (5.61)	1669 (27.45)^a^	274 (4.51)	857 (14.09)	2174 (35.75)
Portal D	4674 (76.86)^a^	207 (3.40)	165 (2.71)	499 (8.21)	145 (2.38)^b^	391 (6.43)
Portal E	238 (3.91)	255 (4.19)	622 (10.23)^b^	166 (2.73)	3956 (65.06)^a^	842 (13.85)
Portal F	65 (1.07)	864 (14.21)	84 (1.38)^b^	36 (0.59)	4441 (73.03)^a^	585 (9.62)
Portal G	3925 (64.55)^a^	541 (8.90)^b^	239 (3.93)	109 (1.79)	157 (2.58)	1113 (18.3)
Portal H	4129 (67.90)^a^	347 (5.71)	681 (11.20)	297 (4.88)	49 (0.81)^b^	572 (9.41)
Portal I	584 (9.60)	663 (10.9)^b^	3537 (58.16)^a^	209 (3.44)	222 (3.65)	870 (14.31)
Portal K	851 (13.99)^b^	91 (1.5)	4020 (66.11)^a^	321 (5.28)	122 (2.01)	666 (10.95)

^a^ Hospital with the lowest RAMR.

^b^ Hospital with the highest RAMR.

### Were Respondents’ Hospital Choices Associated With Particular Information Design Elements?

#### Overview

To explore whether respondents’ hospital choices were influenced by the design, we asked the following questions:

1. Did respondents who used websites that included a given feature choose the hospital with the lowest RAMR significantly more or less often than respondents using portals not including these features?
2. How did respondents who used the portals including this feature rate the overall comprehensibility of the site?
3. Based on their answers to the open-ended question about reasons for their decision, did respondents regard this design feature as useful (appreciative comments) or confusing (critical comments)?

#### Table Without Symbols

Two of 10 portals presented tables without symbols (Table 3). Respondents using these portals chose the hospital with the lowest RAMR significantly less often than respondents using other portals did (46.18%, 575/1245 vs 64.50%, 3119/4836, *P*<.001). They also rated the comprehensibility of the presentation significantly lower (mean 3.07, SD 1.85 vs mean 3.77, SD 1.92; Table 5). This corresponded with negative comments about tables given in response to the open-ended question. Most responses to the open-ended question that mentioned tables without symbols were disapproving:

I cannot decide as the table is not understandable even after reading the explanations.

I think this table is very confusing; it is difficult to interpret the various measures.

This presentation is much too incomprehensible; therefore, I cannot choose 1 of the hospitals.

**Table 5:** Choice of the hospital with the lowest risk-adjusted mortality rate (RAMR).

Information presentation feature	Feature included			Feature not included			Choice of lowest RAMR		Comprehensibility	
	Respondents, n	Selected hospital with the lowest RAMR, n (%)	Comprehensibility,^a^ mean (SD)	Respondents, n	Selected hospital with the lowest RAMR, n (%)	Comprehensibility, ^a^ mean (SD)	χ^2^ (*df*)	*P*	*t* (*df*)	*P*
Table without symbols	1245	575 (46.18)	3.07 (1.85)	4836	3119 (64.50)	3.77 (1.92)	139.2 (1)	<.001	-11.657 (6079)	<.001
Table with symbols	1787	928 (51.93)	3.58 (1.84)	4294	2766 (64.42)	3.65 (1.96)	82.5 (1)	<.001	-1.300 (6979)	.19
Bar chart without symbols	608	392 (64.47)	2.99 (1.84)	5473	3302 (60.33)	3.70 (1.92)	3.9 (1)	.047	-8.626 (6079)	<.001
Bar chart with symbols	2441	1799 (73.70)	4.11 (1.92)	3640	1895 (52.06)	3.31 (1.86)	286.9 (1)	<.001	16.289 (6079)	<.001
Bar chart with traffic light symbols	1814	1341 (73.93)	4.25 (1.93)	4267	2353 (55.14)	3.36 (1.86)	188.3 (1)	<.001	16.774 (6079)	<.001
Bar chart with thumb symbols	627	458 (73.05)	3.70 (1.83)	5454	3236 (59.33)	3.62 (1.94)	44.4 (1)	<.001	1.030 (6079)	.30
Providers ranked by performance	1221	883 (72.32)	4.29 (1.91)	4860	2811 (57.84)	3.46 (1.89)	85.8 (1)	<.001	13.620 (6079)	<.001
Explicit statement about whether higher or lower values indicate better performance	3017	2079 (68.91)	3.85 (1.97)	3064	1615 (52.71)	3.41 (1.86)	167.3 (1)	<.001	9.112 (6079)	<.001
No statement about scale direction, but range for good quality presented	1220	884 (72.46)	4.04 (1.91)	4861	2810 (57.81)	3.52 (1.92)	87.8 (1)	<.001	8.440 (6079)	<.001
*Incomplete data (N/A labels)	2445	1212 (49.57)	3.32 (1.88)	3636	2483 (68.30)	3.84 (1.93)	214.2 (1)	<.001	-10.436 (6079)	<.001

^a^ Based on a 7-point Likert scale with a range of 1=not at all comprehensible to 7=very comprehensible.

#### Table With Symbols

**Figure 2:**
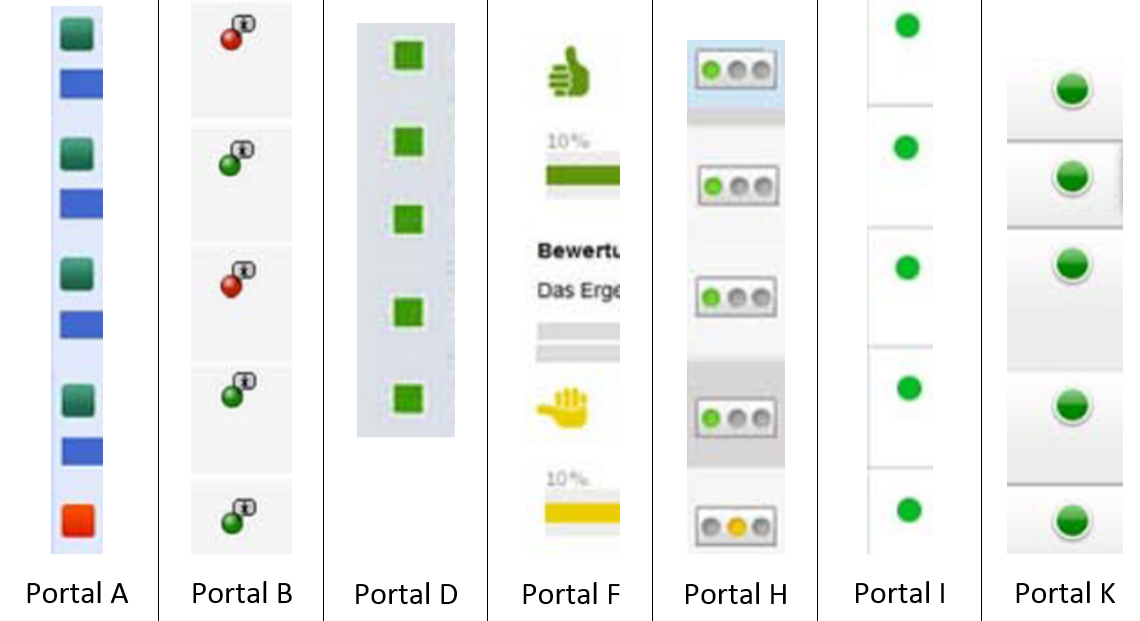
Symbols used by 7 of the 10 portals.

Three of 10 portals presented tables with evaluative symbols (Table 2). Respondents using these portals chose the hospital with the lowest RAMR significantly less often than respondents using other portals (51.93%, 928/1787 vs 64.42%, 2766/4294; *P*<.001) and rated the comprehensibility of the presentation lower (without meeting statistical significance) (Table 5). This corresponded to mainly negative comments about tables and positive ones about symbols given in response to the open-ended question (Table 6). In combination with tables or bar charts, 6 of 10 portals (Table 5) used green, yellow, or red symbols; 1 (Portal F) presented thumbs (thumbs up or thumbs down) symbols in these colors (see Figure 2). Red (3/51) and yellow (4/51) symbols were displayed less often than green ones (44/51, 86%). Three portals presented green symbols for all 5 displayed hospitals.

**Table 6:** Responses to the open-ended question about information presentation.

Information presentation feature	Responses, n
Table without symbols	39 (38 incomprehensible)
Table with symbols	Helpful green symbol: n=59; table: n=36 (35 incomprehensible)
Bar chart without symbols	25 (23 incomprehensible)
Bar chart with symbols	Symbol helpful: n=79; bar chart (incomprehensible): n=39; bar chart (helpful): n=24
Bar chart with traffic light symbols	Bar chart: n=50 (30 incomprehensible, 20 helpful); symbol: n=30 (28 helpful)
Bar chart with thumb symbols	Helpful thumb: n=51; bar chart (incomprehensible): n=9; bar chart (helpful): n=4
Providers ranked by performance	Ranking (helpful): n=3
Explicit statement about whether higher or lower values indicate better performance	Higher values as a reason for hospital choice: n=67
No statement about scale direction, but range for good quality presented	—
Incomplete data (N/A labels)	Complaints about incomplete or missing data: n=59

#### Bar Chart Without Symbols

**Figure 3:**
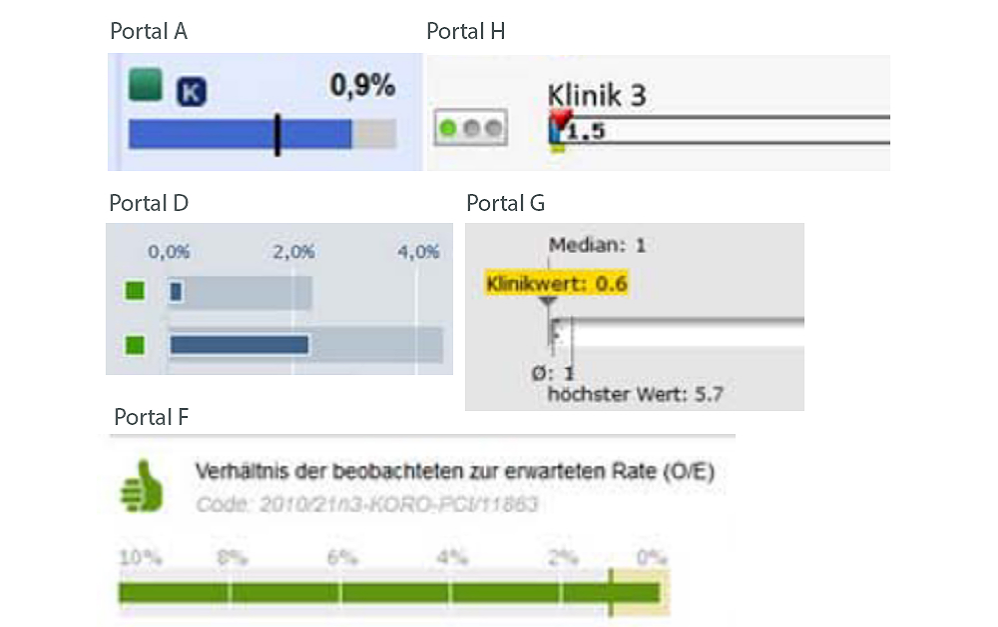
Bar chart presentation taken from 5 portals.

One report card used bar charts without symbols (Table 3 and Figure 3). Respondents using this portal chose the hospital with the lowest RAMR significantly more often than respondents using other portals (64.5%, 392/608 vs 60.3%, 3302/ 5473; *P*=.047), but rated the comprehensibility of the presentation lower (mean 2.99, SD 1.84 vs mean 3.70, SD 1.92; *P*<.001) (Table 5). This corresponded to mostly negative comments about bar charts given in response to the open-ended question (Table 6).

The bars displayed on Portals G and H were too narrow for evaluation. Portal G presented 4 numbers (benchmarking information, median, mean, highest mortality rate for the hospital) in the bar chart, which may have led to information overload. All bar charts in the German portals used longer bars to indicate lower quality, which led to some confusion.

Most responses to the open-ended question that mentioned bar charts without symbols were disapproving:

Diagram is unclear and incomprehensible.

According to this graphic, I would choose clinic 1, even without really knowing which one is better. The graphics are not very useful.

It has the lowest value, but I find the charts very difficult to understand because I cannot work out what they mean.

#### Bar Chart With Symbols

Four of 10 portals used bar charts with symbols (Table 2 and Figure 2). Respondents using these portals chose the hospital with the lowest RAMR significantly more often than respondents using other portals (73.70%, 1799/2441 vs 52.06%, 1895/3640; *P*<.001) and rated the comprehensibility of the presentation higher (mean 4.11, SD 1.92 vs mean 3.31, SD 1.86; *P*<.001) (Table 5). Comments about symbols were mostly positive, but comments about bar charts were mixed (Table 6). Respondents testing Portal A (bar chart with symbols) referred to the bar chart with a blue bar for their decision (see Figure 2), which attracted 10 approving comments, such as:

I didn’t rely on percentages, but the longest blue bar in the diagram.

Longest bar and lowest result.

But, more comments (n=19) were disapproving, such as:

What is the meaning of the bar—is a lot of blue good?

0.9% is the smallest number, even if the blue bar is the longest.

What does this blue bar mean?

#### Bar Chart With Traffic Light Symbols

Three of 10 portals used bar charts with traffic light symbols (Table 3 and Figure 2). Respondents using these portals chose the hospital with the lowest RAMR significantly more often than respondents using other portals (73.70%, 1341/1814 vs 52.06%, 2353/4267; *P*<.001); they also rated the comprehensibility of the presentation higher (mean 4.25, SD 1.93 vs mean 3.36, SD 1.86; *P*<.001) (Table 5). Comments about symbols were mostly positive, but comments about bar charts were mixed (Table 6).

#### Bar Chart With Thumb Symbols

One of 10 portals used bar charts with thumb symbols (Table 3 and Figure 2). Respondents using this portal chose the hospital with the lowest RAMR significantly more often than respondents using other portals (73.05%, 458/627 vs 59.33%, 3236/5454, *P*<.001), but assigned a similar rating to the comprehensibility of the presentation (mean 3.70, SD 1.83 vs mean 3.62, SD 1.94; *P*=.30) (Table 5). Comments about symbols were positive, but the few comments about bar charts were mixed (Table 6).

#### Ranking Providers by Performance

Two of the 10 portals ranked providers by performance (Table 2 and Figure 2). Respondents using these portals chose the hospital with the lowest RAMR significantly more often than respondents using other portals (72.32%, 883/1221 vs 57.84%, 2811/4860; *P*<.001) and rated the comprehensibility of the presentation higher (mean 4.29, SD 1.91 vs mean 3.46, SD 1.89; *P*<.001) (Table 5).

#### Explicit Statement That Low Values Indicate Good Performance

Five of 10 portals explicitly stated that lower values indicated better performance (Table 3). Respondents using these portals chose the hospital with the lowest RAMR significantly more often than respondents using other portals (68.91%, 2079/3017 vs 52.71%, 1615/3064; *P*<.001) and rated the comprehensibility of the presentation higher (mean 3.85, SD 1.97 vs mean 3.41, SD 1.86; *P*<.001) (Table 5).

#### No Explicit Statement About Scale Direction, But “Good Quality” Range Identified

Two of 10 portals explicitly gave a “good quality” range (Table 3 and Figure 2). Respondents using these portals chose the hospital with the lowest RAMR significantly more often than respondents using other portals (72.46%, 884/1220 vs 57.81%, 2810/4861; *P*<.001) and rated the comprehensibility of the presentation higher (mean 4.04, SD 1.91 vs mean 3.52, SD 1.92; *P*<.001) (Table 5).

#### Incomplete Data (N/A as a Value)

Four of the 10 portals presented incomplete data (N/A as a value) (Table 3) for 1 or more measures (confidence intervals, frequency of cases treated, frequency of mortality, mortality rates, comments about hospital or quality controlling agency). Respondents using these portals chose the hospital with the lowest RAMR significantly less often than respondents using other portals (49.57%, 1212/2445 vs 68.30%, 2483/3636; *P*<.001) and rated the comprehensibility of the presentation lower (mean 3.32, SD 1.87 vs mean 3.84, SD 1.93; *P*<.001) (Table 5). A total of 59 responses to the open-ended question complained about incomplete or missing data (Table 6). Comments of respondents about missing values on Portal B (hospitals 4 and 5 had missing data in a number of cases; hospital 5 had the lowest RAMR) included:

As I do not know how many cases hospital 4 or 5 have, I decided against them.

Hospitals 4 and 5 seem to be suspect as they do not show the frequency of cases as the basis for the observed to expected rate.

Because the 2 remaining best-practice elements, evaluative word labels and highlighting best providers, were not included in any of the report cards we studied, we were not able to not investigate their effect on hospital choice.

## Discussion

### Overview

The 3 research questions answered are as follows:

1. What information presentation elements were utilized?
2. Which led to better comprehension?
3. Which information presentation elements had similar effects to those reported in the evidence-based recommendations described in the literature?

### What Information Presentation Elements Were Utilized?

We identified 10 elements that were used in the presentation of RAMR for coronary catheterization by 10 German portals (see Table 2).

### Which Led to Better Comprehension?

Report cards using the following 7 presentation elements were more comprehensible, because respondents choose the hospital with the lowest RAMR significantly more often: (1) bar chart without symbols, (2) bar chart with symbols, (3) bar chart with traffic light symbols, (4) bar chart with thumb symbols, (5) providers ranked by performance, (6) an explicit statement about whether higher or lower values indicated better performance, and (7) no statement about scale direction, but a presented range for good quality.

Furthermore, respondents rated presentations as more comprehensible when they contained the following 4 elements: (1) bar chart with symbols, (2) bar chart with traffic light symbols, (3) providers ranked by performance, (4) explicit statement about whether higher or lower values indicated better performance, and (5) no statement about scale direction, but range for good quality presented.

Report cards using the following 2 presentation elements were less comprehensible, because respondents using these elements choose the hospital with the lowest RAMR significantly less often. This was true for tables without symbols or for incomplete data (N/A labels). Moreover, respondents rated presentations as less comprehensible when they contained tables without symbols or incomplete data (N/A labels).

### Which Information Presentation Elements Had Similar Effects to Those Reported in the Evidence-Based Recommendations Described in the Literature?

Based on the 13 identified international reports about the presentation of information on report cards, 14 information presentation elements were identified. These studies relied on a variety of methods, including in-depth or cognitive interviews and focus groups, plus online, telephone, or paper-based surveys as well as controlled laboratory experiments (see Multimedia Appendix 1). This variety of methods made it possible to obtain insights from different perspectives about the behavior of users, but also made it more difficult to systematically compare the results. Additionally, samples used in the studies varied widely; sizes ranged from 59 to 2052. Sample selections also had limitations, such as the use of convenience samples, limited geographic locations, low response rates, or a possible selection bias because of questioning by mail and Internet. These limitations have to be taken into account when conclusions are drawn from those studies.

### Did the Respondents to Our Survey Make Choices and Give Reasons That Corresponded to the Results of the 13 Identified International Reports?

Our respondents, like those in the study by Gerteis et al [29], made more interpretive errors with the numeric formats (table without symbols) than with the graphical formats (bar charts). They had more difficulty understanding numeric tables than other presentation formats, a finding similar to that of Donelan et al [31].

Similar to Geraedts et al [30], we observed that the hospital with the lowest mortality was chosen more often when tables with symbols were used than when tables without symbols were used. In our survey, tables with symbols were rated better for comprehension (mean 3.58, SD 1.84) than bar charts without symbols (mean 2.99, SD 1.84). However, we cannot fully endorse Gerteis et al’s [29] recommendation (in a study limited to 90 respondents) that tables with symbols should be used rather than a standard bar chart without symbols. In our survey, bar charts without symbols more frequently resulted in the choice of the hospital with the lowest mortality (64.47%) than tables with symbols (51.93%), but as discussed previously, missing data rather than the presentational format may have been the major reason.

Similar to Damman et al [32], we found that bar charts were used quite often and, like Gerteis et al [29], we found that graphical formats (standard bar chart without symbols) were not liked by respondents. However, we did not confirm the findings of Geraedts et al, who presented the formats of presentations to physicians, that bar charts without symbols did not assist the comprehension of data on hospital quality [30] or were the format least well understood by participants [29]. In our sample, more respondents who used a bar chart without symbols (64.47%) chose the hospital with the lowest mortality rate than respondents who did not (60.33%). As Figure 3 shows, Portal G displayed bars that were too narrow for evaluation, but numbers were also included. Thus, respondents may still have been able to evaluate the presentation by evaluating the numerical value instead of using the bar chart (“It has the lowest value, but I find the charts very difficult to understand”).

The respondents in our sample who used portals that displayed bar charts with symbols were more likely than average to choose the hospital with the lowest mortality rate, a result which is in accordance with other studies [31-33]. Two portals ranked hospitals by performance, as recommended in several studies [12,15,30,33,35,36]. This was also valued by our participants.

Our results support the recommendation that whether high or low values indicate good performance should be explicitly stated [8,36]. Incomplete data (N/A given as a value) had a negative influence on provider assessment, as Gerteis et al [29] suggested.

To summarize, 5 of 14 information presentation elements were found to be associated with better comprehension as reported in literature: (1) bar charts with symbols, (2) explicit statement about whether higher or lower values indicated better performance, and (3) providers ranked by performance. A reduction of user comprehension was associated with (4) incomplete data (N/A labels) and (5) tables without symbols, again in accordance with literature-based evidence. Reasons have been given for the 2 discrepant findings on (6) bar charts without symbols (improvement of comprehension observed in our German survey, but not described in international literature) and (7) tables with symbols (improvement of comprehension described in international literature, but not observed in our German survey).

### Conclusions

To our knowledge, this is the first study to systematically analyze the most commonly used public report card designs in Germany. It is also the first study to analyze German report cards using real-world applications [15] instead of controlled laboratory studies. The best-practice evidence found in 13 international studies led to 14 findings about information presentation elements. However, due to limitations of these studies, conclusions have to be drawn carefully. Five of these findings were in agreement with our findings about German report card designs: (1) avoid tables without symbols, (2) include bar charts with symbols, (3) state explicitly whether high or low values indicate good performance or provide a “good quality” range, (4) avoid incomplete data (N/A given as a value), and (5) rank hospitals by performance. However, due to limitations of our study as described subsequently, these recommendations are preliminary and should be subject to further evaluation.

The implementation of 4 of these findings should not present an insurmountable obstacle to public reporting instruments because they can be achieved by redesigning the format. However, ranking hospitals by performance may present substantial difficulties because ordering by performance is often resisted by providers [7]. Ordering makes report sponsors responsible for determining what constitutes a meaningful difference in mortality rates. The benchmarking information available in Germany might provide a basis for ranking providers. However, ranking might also require measures of statistical significance to be provided that have the potential to confuse users [8,31,36,50,51].

Externally validated measures of hospital quality could also be used as the basis for ranking hospitals and are readily available in Germany [17]; indeed, most German portals use these results to assign their traffic light symbols. However, because only approximately 0.1% of quality measures are finally validated as being qualitatively discrepant, the ranking of hospitals in these terms is difficult. As a respondent put it: “All the symbols for hospital quality are green.”

### Limitations

Several limitations are relevant for this study. This report only investigated the design of report cards. Other important features of report cards (eg, the quality of explanations, use of technical terms, and provision of a quality framework) will be the subject of further studies. Respondents in our study only saw a small part of the public reporting portals because we focused on the presentation of 1 RAMR measure. Five report cards (Portals B, E, F, G, I) did not allow a performance comparison of hospitals in the way we presented it in this study, but were adapted to enable respondents to make the comparison we were investigating. We limited our search for recommendations on best practice in the presentation of information in public report cards to the PubMed and Cochrane medical databases and did not take into account other databases, such as PsychINFO.

Because our study was designed as a Web survey, the results presented might be influenced by self-selection of the study participants. Better-educated persons were overrepresented. Because education levels influence comprehension of public report cards, this might have influenced our results; certain features of presented information might have been misunderstood depending on the education of respondents. Hibbard et al [52] stated that higher education levels were related to improved performance. Emmert et al [53] found that those with a higher level of education tended to comprehend public reporting better. Damman et al [32] stated that consumers’ educational level was positively related to the correct interpretation. Donelan et al [33] found that respondents with at least some college education were significantly more likely to identify surgeons with the lowest risk-adjusted mortality, compared with respondents having no college education.

## Multimedia Appendix 1

Methodological quality of used literature.

## Multimedia Appendix 2

Screenshots of report cards.

## Abbreviations

AGOF: Arbeitsgemeinschaft Online Forschung
PCI: percutaneous coronary intervention
RAMR: risk-adjusted mortality rate
