# Supplementary material for: Information Presentation Features and Comprehensibility of Hospital Report Cards: Design Analysis and Online Survey Among Users
Source: J Med Internet Res. 2015 Mar 16;17(3):e68. doi: 10.2196/jmir.3414 (PMC4381815; doi:10.2196/jmir.3414)
Supplement: Supplementary file 1 [file jmir_v17i3e68_app1.pdf]

Authors (year)	Country	Study design/sample size	Setting	Results	Implications for presentation (for our article)	Study limitations
7] Hibbard et al. 2010)	USA	Report on best practice in public reporting	Different findings put together to a guideline.	PR instruments are often confusing for consumers because they use too many dimensions of information or display it in a way that leads users to misinterpret the data shown.	- It should be explicitly stated whether high or low values indicate good performance, regardless of the direction of the scale.	- This paper is a guideline about the way quality data should be reported/presented to consumers, reflecting the state of the art. There is no systematic review within the paper, so one limitation might be that a depiction of the state of art is missed.
1] Faber et al. 2009)	USA	Systematic Review		All 14 studies included examine quality information, usually Consumer Assessment of Health Care Providers and Systems (CAHPS), with respect to its impact on the consumer's choice of health plans. Easy-to-read presentation formats and explanatory messages improve knowledge about and the attitude towards the use of quality information; however, the weight given to quality information depends on other features, including free provider choice and costs. In real-world settings, having seen quality information is a strong	- Providers should be ranked by performance.	- Small number of studies because of the stringent inclusion criteria.<br>- Most included studies assessed the use of quality information within a health plan setting; results based on health plan choice will not match those of provider choice.

				determinant of choice of higher quality-rated health plans.		
.4] Hibbard, eters (2003)	USA	Literature Review	Review of what is known from studies of human judgment and decision-making, and discussion of the implications of supporting informed consumer choice based on health care information.	Comprehension, motivation, and the actual use of the information are increased when cognitive effort is reduced, when the decision-maker is moved closer to the actual experience, and when the meaning of information is highlighted for the decision-maker. Also, the characteristics of the audience receiving the information should be considered (e.g., younger vs. older or highly-educated vs. less-educated people).	- Ranking by performance increased the frequency of users choosing higher-performing services.	
28] Gerteis et al. 2007)	USA (Boston, McLean)	One-to-one personal interview (N = 90)	Seven different evaluative, numeric, and graphic templates were developed and used within the study. Respondents were asked to choose a nursing home for a fictitious relative who was discharged from hospital soon after suffering a stroke. Each respondent reviewed all seven templates.	Fewer than half of the respondents accurately interpreted bar graphs as currently displayed on the Nursing Home Compare Web site. Respondents made fewest errors on templates that used words to characterize performance as better, average, or worse.	- Consider using a table design such as the "evaluative table with stars" rather than a bar chart.<br>- Evaluative tables using words or stars are superior to numeric tables.<br>- Standard bar charts were not well-liked by respondents and led to the lowest levels of comprehension.<br>- Numeric tables and bar charts often led respondents to conclude that the worst performing nursing homes (those with the higher	- Sample was limited to 90 respondents from two geographical locations.<br>- Only CMS reported nursing home quality measures.<br>- Only a few templates were designed to represent three types of reporting formats.<br>- Limited explanatory text within the data displays.<br>- Due to the study setting, some of the errors reported may reflect respondents misunderstanding of interview questions rather

					percentages) were the best, notwithstanding the warning label at the top.<br>- Incomplete data (missing values) have a negative influence on provider assessment and have the potential to influence the decision.	than misinterpretation of the data displays.
29] Donelan et . (2011)	USA (Massachusetts)	Online-Survey (N = 337)	Respondents viewed four displays of cardiac surgeon ratings and were asked which surgeon they would be most and least likely to choose. Further questions focused on public reporting in general as well as on information on demographics and health care utilization.	Accurate identification of best surgeon performance varied by display format, with a high of 66% accuracy for one display and a low of 16% for another. Only 6.4% identified the surgeon with the lowest mortality risk across all four displays. Respondents with at least some college education were significantly more likely to identify the surgeon with the lowest risk-adjusted mortality than respondents with no college education. In one display, the surgeon with the lowest risk-adjusted mortality was effectively penalized for taking on higher-risk patients; respondents tended to select the surgeon with the lowest-risk population but the highest risk-adjusted mortality. Overall, 82% of respondents said that access to these types of data	- Graphic displays were more helpful to users than text-only tables.<br>- Symbols and bar charts should be used.<br>- Participants liked to use symbols to identify the best surgeon.	- Cross-sectional view of the response to report cards, but no conclusive study.<br>- Risk of sample response bias through questioning by mail and internet.<br>- The circumstances faced by the consumers while in the experiment differed from real-life.<br>- No distinction between effects of data presentation and consumer preferences was possible.

				would be "absolutely essential" or "very important" in choosing a surgeon.		
[illegible]0] Geraedts et [illegible]. (2012)	Germany	Computer-Assisted Telephone Interview (N = 300)	Eight presentation formats were shown to non-hospital-based physicians in the northern, southern, and eastern parts of Germany. Physicians had to decide.	Physicians' ratings of the formats differed significantly. Formats combining numeric information and evaluative cues performed best in terms of information content, comprehensibility, and preference. Comprehension of presentation formats also differed. Even though physicians' accuracy of interpreting "Simple Star Rating" was best (94%), the majority of participants accepted only formats that contained detailed numerical information ("Ranked Numeric Table"; 91%).	- Physicians preferred formats that used traffic light symbols to code the value of indicators (numeric table with traffic lights).<br>- Comprehension was lowest when data was presented in bar charts.<br>- Physicians preferred formats that used symbols, e.g., traffic lights.<br>- Physicians prefer presentation formats that combine individual indicator values with evaluative features such as rankings.	- Focused on the typically used formats and did not test the full range of presentation formats.<br>- Only a reduced number of presentation formats were presented to each participant.<br>- No systematic variation of the attributes and their combinations.<br>- Participants were queried about a hypothetical counseling scenario and may have answered differently in an actual counseling situation.
[illegible]1] Damman et [illegible]. (2011)	Netherlands	Online-Survey (N = 2052)	Respondents filled out an online questionnaire at home. They viewed four randomly chosen formats out of a total of 15 formats. Subsequently, they were asked to answer questions	Both presentation features and consumer characteristics (age and education) significantly affected consumers' responses. Formats using combinations of bar charts and stars, three stars, an alphabetical ordering of providers, and no inclusion of a global rating supported consumers. The effect of the features of the presentation differed across the outcome	- Bar charts were commonly used (43% of public reporting websites).<br>- A combination of bar charts and star ratings facilitated correct interpretation by users.<br>- When providers were ordered alphabetically participants were significantly more likely to	- Very low response rate.<br>- Less educated people often did not complete the questionnaire, underlining that the information and/or questions were difficult for consumers to understand.<br>- No investigation of differences between consumer subgroups, e.g., more or less numerate

			about the information in these formats in order to choose the best-performing providers.	variables.	make effective use of the data (i.e., choose the best provider) than when providers were ordered by performance.	individuals.
32] Hibbard et . (2001)	USA (Eugene and Springfield, Oregon; University of Oregon)	Paper-Survey (elderly 65+ Medicare sample, N = 253; nonelderly <65 sample, N = 239)	Participants were asked to review information and complete a series of decision tasks involving the use of comparative information and making health plan selections.	The findings indicate that there are approaches to data presentation that help consumers who have lower skills to use information more accurately. Some of these presentation strategies (for example, relative stars) improve comprehension among the less skilled, and other strategies (e.g., evaluative labels) appear to aid those in the midrange of comprehension skills.	- Adding stars to bar charts increases comprehension significantly.<br>- Adding evaluative labels to bar charts did not increase comprehension.<br>- Ranking plans by performance significantly decreased errors in interpreting data.<br>- One of the more powerful display strategies is to rank providers in terms of performance.	- Information presented to the participants was contrived and only partially mirrored real-world situations.<br>- Participants were not random samples but convenience samples, which are not generally representative of the larger population.<br>- Nonelderly were more highly educated than the general population.<br>- Information shown was less complex and less ambiguous than the information which is actually disseminated in real world.
33] Mazor, odd (2009)	USA (Central Massachuset ts)	In-depth interviews (qualitative descriptive study) (N = 59)	Participants had to pass three different parts of the interview. The first part focused on print	Many interviewees were unfamiliar with health care associated infections (HAIs) and were distressed to learn that HAIs occur and can result in death. Public	- Color-coding important information improves comprehension.<br>- Providers should be ranked in descending order of	- Participants were drawn from a limited geographical area and were relatively homogenous in terms of

			reports, the second part focused on improved reports due to the responses to the versions used in the first part, and in the third part participants viewed some existing Web-based reports in real time. In each part the interviewees had to find out the "best" hospital according to the information provided.	reporting was seen as unlikely to affect hospital choice; other factors were considered more influential. Interviewees recommended that reports be brief and include information on prevention as well as performance data. For public reporting of HAIs to be successful, it is necessary to pay attention to report content and format. Consumer involvement can help identify potential sources of confusion and methods of improving reporting.	quality, as this was valued by participants and increased their comprehension.<br>- It should be stated explicitly whether high or low values indicate good performance, regardless of the direction of the scale.	race and ethnicity.<br>- Interviewees tended to be more highly educated than the state average for Massachusetts.<br>- Sample not large enough to estimate prevalence or frequencies of particular views, or to examine differences associated with consumer characteristics.<br>- Low number of interviewees for each set of materials.
[illegible]4] Peters et al. [illegible]2007)	USA (Oregon)	Randomized Survey (N = 303)	Participants had to answer questions concerning comprehension and choice of high-quality hospitals after passing a numeracy test which showed people's ability to convert (e.g., percentages to proportions, proportions to percentages, etc.)	Results of three studies support the idea that "less is more" when presenting consumers with comparative performance information to make hospital choices. Results were particularly strong for those with lower numeracy, who had higher comprehension and made better choices when the information-presentation format was designed to ease the cognitive burden and highlight the meaning of important information.	- Only important information should be made easier to evaluate using symbols.<br>- Comprehension of respondents with low numeracy was significantly improved in the ordered compared to the unordered condition.<br>- Performance data should be displayed such that high values always represent high performance.	- Convenience sample of employed adults.<br>- Measuring numeracy as done in this study is not always possible because of limitations of time and phone surveys, and the anxiety engendered by a math test in some people.
[illegible]7] Hibbard et	USA	Controlled	Participants had to	Some presentation approaches	- Stars-only formats should be	- Decisions made due to the

. (2002)	(Oregon)	laboratory experiment (N = 162)	answer four different parts of the experiment, which had different targets using within-subject designs or between-subject designs.	make it easier for users to process and integrate quality data into their choices (visual cues, ordering on performance, provide trend data). However, other presentation formats influence consumers' decisions in ways that undermine their self-interest (summarizing data).	used in preference to numerical values.	research questions only partially mirror real-world decisions.<br>- Laboratory conditions may not capture participants' actual behavior.<br>- Study does not address the question of general acceptance of comparative reports.<br>- Generalizability of findings difficult because of use of a convenience sample.
8] Uhrig et al. 006)	USA (Oregon and North Carolina)	Randomized experiment (N = 152)	Participants were randomly assigned to three different study groups and were asked within the experiment to choose a Medicare plan based on the information given in materials which were handed to them.	Assessment of the efficacy of materials that integrated comparative information on cost, benefits, and quality for employer-based retiree health plans and Medicare Advantage plans in a randomized experiment to test the impact of content and format. Results indicate that older consumers who received the intervention materials found the materials easier to us, gained greater knowledge about Medicare from them, were more likely to value comparative quality information, were more likely to select higher quality plans, and	- Highlighting information about quality results in greater understanding by participants.	- No random, probability-based sample.<br>- Hypothetical plan choices with no real consequences of the choices made.<br>- Medigap plans could not be included because there is no quality information available.<br>- Participants were handed the intervention materials.<br>- No obvious choice for control materials.

				were more likely to choose a plan that reflected the dimensions they found most important compared to older consumers receiving the control materials.		
39] Harris-[illegible]ojetin et al. 2001)	USA (Portland, Washington DC, Baltimore, Raleigh/ Durham, Wichita, Kansas City)	Focus Groups, Cognitive Interviews (N = 268)	Participants in focus groups and cognitive interviews had to answer questions on the materials sent to them prior to the sessions. The materials contained information about both the quality of care and the costs of different health plans.	A method is suggested to help consumers narrow their plan choices by breaking down the process into smaller decisions using a set of guided worksheets.	- Presentation formats that highlighted key messages increased comprehension.	- Focus groups and cognitive interviews were performed in a controlled environment. Results may differ from the findings under real-world conditions.<br>- Focused on plan-level comparisons and did not consider overlapping networks.<br>- Findings are not generalizable to a larger population, but provide a rich data source of topics for further investigation.
