# Supplementary material for: Information Presentation Features and Comprehensibility of Hospital Report Cards: Design Analysis and Online Survey Among Users
Source: J Med Internet Res. 2015 Mar 16;17(3):e68. doi: 10.2196/jmir.3414 (PMC4381815; doi:10.2196/jmir.3414)
Supplement: Supplementary file 2 [file jmir_v17i3e68_app2.pdf]

Portal A

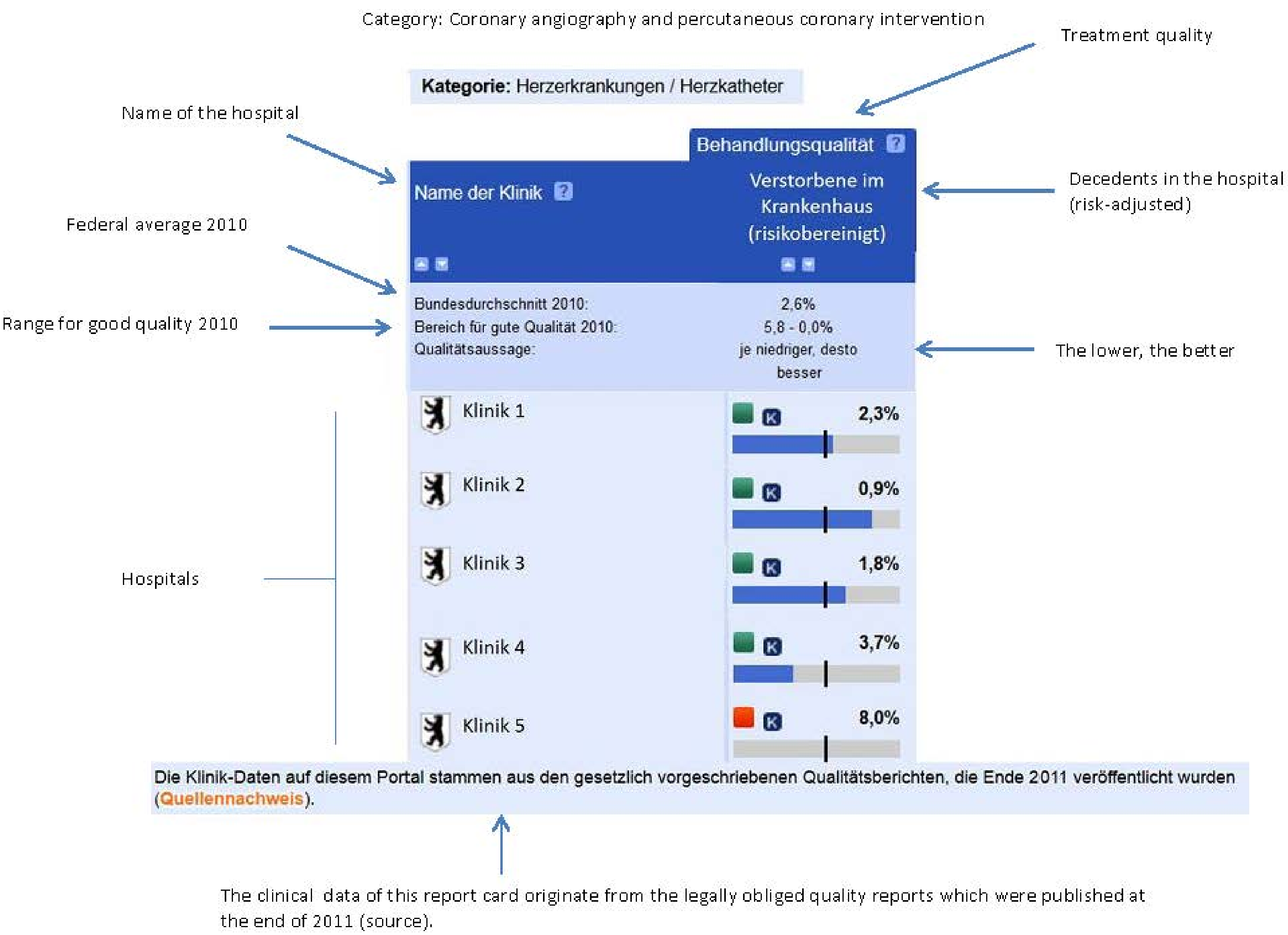

Category: Coronary angiography and percutaneous coronary intervention
Treatment quality
Kategorie: Herzerkrankungen / Herzkatheter
Name of the hospital
Behandlungsqualität
Name der Klinik
Verstorbene im Krankenhaus (risikobereinigt)
Decedents in the hospital (risk-adjusted)
Federal average 2010
Range for good quality 2010
Bundesdurchschnitt 2010: 2,6%
Bereich für gute Qualität 2010: 5,8 - 0,0%
Qualitätsaussage: je niedriger, desto besser
The lower, the better
Hospitals
Klinik 1 2,3%
Klinik 2 0,9%
Klinik 3 1,8%
Klinik 4 3,7%
Klinik 5 8,0%
Die Klinik-Daten auf diesem Portal stammen aus den gesetzlich vorgeschriebenen Qualitätsberichten, die Ende 2011 veröffentlicht wurden (Quellennachweis).
The clinical data of this report card originate from the legally obliged quality reports which were published at the end of 2011 (source).

Portal B

Die hier veröffentlichten Qualitätsindikatoren sind Hilfsmittel, die es ermöglichen sollen, Qualität zu messen und zu bewerten. Dabei wird die Qualität nicht direkt gemessen, sondern durch Zahlen bzw. Zahlenverhältnisse indirekt abgebildet. Dieses kann dazu zu führen, dass der Ergebniswert des Krankenhauses rechnerisch als auffällig markiert wird, inhaltlich der Wert aber als unbedenklich einzustufen ist. Leider fehlt in den Ergebnissen der Krankenhäuser sehr häufig die Angabe der für das Ergebnis verantwortlichen Fallzahl, so dass hier eine qualitative Prüfung der Ergebniswerte nicht möglich ist. Die farbliche Markierung der Qualitätsindikatoren stellt lediglich die rechnerische Interpretation der Ergebniswerte des Krankenhauses dar. Für sämtliche Angaben zu den Qualitätsindikatoren wird keine Haftung übernommen.

Information that the published quality indicators on this page enable to measure and evaluate quality. Thereby, quality is not measured directly but rather presented by means of numbers/ratios. This can lead to a result of a hospital which is marked as discrepant, but the result is marked as non-discrepant.
Further information is given, that the record of the total number of cases is missing very often. For this reason, a qualitative monitoring of the results is not possible.
Colors are used to indicate the computational interpretation of the results.

Die Leistungsbereiche im Detail:

- Behandlung von Brustkrebs
- Entfernung der Gallenblase
- Geburtshilfe
- Herzkatheteruntersuchung- und behandlung
- Herzschrittmachereinsatz
- Hüftgelenknaher Knochenbruch
- Lungenentzündung
- Wechsel des Kniegelenk-Implantats
- Behandlung von Druckgeschwüren
- Frauenheilkunde
- Halsschlagaderoperation
- Herzschrittmacher-Aggregatwechsel
- Hüftgelenkersatz
- Kniegelenkersatz
- Wechsel des Hüftgelenk-Implantats

Clinical areas in detail:

- Treatment of breast cancer
- Obstetrics
- Etc.

Qualitätsindikatoren

Herzkatheteruntersuchung- und behandlung (Glossar) Details

Ratio of the observed to the expected rate (O/E)

Hospitals	Qualitätsindikator	Status und Ergebnis strukturierter Dialog	Ergebnis	Vetrauensbereich (Glossar)	Referenzbereich (Glossar)	Bundeswert (Glossar)	Fälle Gesamt	Ergebnis-relevante Fälle
Klinik 1	Verhältnis der beobachteten zur erwarteten Rate (O / E) *2010/21n3-KORO-PCI/11863*	red	3.8 %	k.A.	0 - 2.4 %	1.1 %	380	k.A.
Klinik 2	Verhältnis der beobachteten zur erwarteten Rate (O / E) *2010/21n3-KORO-PCI/11863*	green	2.1 %	k.A.	0 - 2.4 %	1.1 %	538	k.A.
Klinik 3	Verhältnis der beobachteten zur erwarteten Rate (O / E) *2010/21n3-KORO-PCI/11863*	red	2.5 %	k.A.	0 - 2.4 %	1.1 %	1175	k.A.
Klinik 4	Verhältnis der beobachteten zur erwarteten Rate (O / E) *2010/21n3-KORO-PCI/11863*	green	2.0 %	k.A.	0 - 2.4 %	1.1 %	k.A.	k.A.
Klinik 5	Verhältnis der beobachteten zur erwarteten Rate (O / E) *2010/21n3-KORO-PCI/11863*	green	0.8 %	k.A.	0 - 2.4 %	1.1 %	k.A.	k.A.

- Status and Structured Quality Dialogue
- Outcome
- Confidence intervall
- Reference range
- Federal average
- Number of cases treated
- Number of cases relevant

*** Erläuterung der Symbole:**

- (green) Das Ergebnis des Krankenhauses liegt im Referenzbereich oder das Sentinel Event ist nicht vorgekommen.
- (yellow) Das Ergebnis des Krankenhauses liegt außerhalb des Referenzbereiches, ist jedoch besser als der Bundesdurchschnitt.
- (red) Das Ergebnis des Krankenhauses liegt nicht im Referenzbereich und ist schlechter als der Bundesdurchschnitt oder das Sentinel Event ist vorgekommen.
- (grey) Das Ergebnis des Krankenhauses ist nicht verfügbar, der gelieferte Wert ist unplausibel oder der bundesweite Referenzbereich ist nicht definiert.

The outcome is within the reference range or no sentinel event was documented.

The outcome is outside of the reference range, but better than the federal average.

The outcome is outside of the reference range and lower than the federal average or a sentinel event was documented.

The hospital outcome is not available, the result is implausible or the federal reference range is not defined.

Portal C

Result presentation of comparison

Ergebnisdarstellung des Vergleichs

Results for selected quality indicators from the procedure QSKH-RL

Ergebnisse für ausgewählte Qualitätsindikatoren aus dem Verfahren der QSKH-RL

Leistungsbereiche

Koronarangiographie und Perkutane Koronarintervention (PCI)

Clinical areas
Coronarangiography and percutaneous coronary intervention (PCI)

Legend
Hospitals

Legende

A Klinik 1
B Klinik 2
C Klinik 3
D Klinik 4
E Klinik 5

Quality indicator of the clinical area coronary angiography and percutaneous coronary intervention

Ratio of the observed to the expected rate (O/E)

Qualitätsindikatoren des Leistungsbereichs Koronarangiographie und Perkutane Koronarintervention (PCI)

	A	B	C	D	E	
**Verhältnis der beobachteten zur erwarteten Rate (O/E)**						
Strukturierter Dialog	1	2	2	9	8	Structured Quality Dialogue
Ergebnis	3,3	2,4	1,5	entfällt	1,8	Outcome
Vertrauensbereich	entfällt	entfällt	entfällt	entfällt	entfällt	Confidence intervall
Referenzwert (Bund)	<=2,4			<=2,4		Federal average
Kommentar des Krankenhauses	Kommentar			Kommentar	Kommentar	Comment hospital
Kommentar/ Erläuterung der auf Bundes- bzw. Landesebene beauftragten Stellen					Kommentar	Comment authority

Für den Fall, dass für einen Qualitätsindikator kein Ergebnis in der Tabelle enthalten ist, kann dies daran liegen, dass dem Krankenhaus (z. B. auf Grund geringer Fallzahl) kein Ergebnis durch den strukturierten Dialog mitgeteilt wurde.

In case that the table does not contain any result for one quality indicator, it can be due to the fact, that the hospital (e.g. less cases) was not communicated the result of the Structured Quality Dialogue.

Strukturierter Dialog

0 **Der Strukturierte Dialog ist noch nicht abgeschlossen. Derzeit ist noch keine Einstufung der Ergebnisse möglich.**
1 **Das Ergebnis wird nach Abschluss des Strukturierten Dialogs als qualitativ unauffällig eingestuft.**
2 **Das Ergebnis wird nach Abschluss des Strukturierten Dialogs als qualitativ unauffällig eingestuft. Die Ergebnisse werden im Verlauf besonders kontrolliert.**
3 **Das Ergebnis wird nach Abschluss des Strukturierten Dialogs als qualitativ auffällig eingestuft.**
4 **Das Ergebnis wird nach Abschluss des Strukturierten Dialogs als erneut qualitativ auffällig eingestuft.**
5 **Das Ergebnis wird bei mangelnder Mitwirkung am Strukturierten Dialog als qualitativ auffällig eingestuft.**
6 **nicht besetzt.**
7 **nicht besetzt.**
8 **Das Ergebnis ist unauffällig. Es ist kein Strukturierter Dialog erforderlich.**
9 **Sonstiges (Im Kommentar erläutert.)**
10 **nicht besetzt.**
11 **nicht besetzt.**
12 **nicht besetzt.**
13 **Das Ergebnis wird nach Abschluss des Strukturierten Dialogs wegen fehlerhafter Dokumentation als qualitativ auffällig eingestuft.**
14 **Das Ergebnis wird nach Abschluss des Strukturierten Dialogs wegen fehlerhafter Dokumentation als erneut qualitativ auffällig eingestuft.**

1) Upon conclusion of the Structured Quality Dialogue, the result is classified as qualitatively non-discrepant.
2) Upon conclusion of the Structured Quality Dialogue, the result is classified as qualitatively non-discrepant. The results will be subject to special monitoring over the further course.
8) The result is classified as non-discrepant. No Structured Quality Dialogue is necessary.
9) Other (explained in the commentary).

See *Aqua Institut (2012)* for a complete description .

Portal D

Quality results regarding heart catheter interventions

Qualitätsergebnisse bei Herzkatheteranwendungen

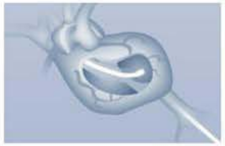

Quality attribute: low number of deaths

Qualitätsmerkmal: Geringe Zahl an Todesfällen

Good quality is considered when the observed rate of deaths is lower or at the same level as the expected rate.

Gute Behandlungsqualität liegt vor, wenn die tatsächliche Rate an Todesfällen im Zusammenhang mit einer Herzkatheteranwendung geringer oder genauso niedrig ist wie die erwartete Rate.

▸ weitere Informationen anzeigen

The following two illustrations illustrate the mortality during a heart catheter intervention.

Die folgenden **zwei Schaubilder** zeigen, wie selten Patienten im Zusammenhang mit einer Herzkatheteranwendung im Krankenhaus sterben:

Ballonkatheterbehandlungen: So häufig ist in Hamburgs Krankenhäusern die tatsächliche Rate an Todesfällen geringer als die erwartete:

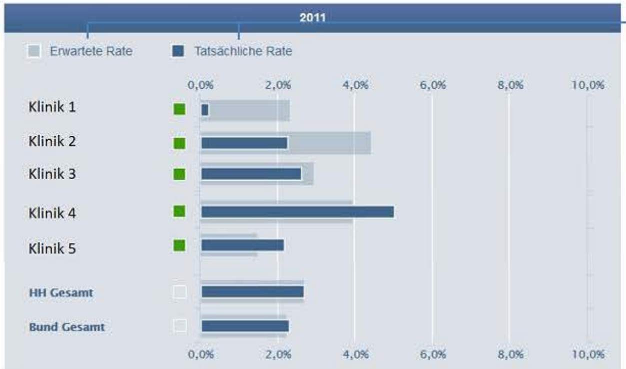

Hospitals

Expected rate

Observed rate

Explanation of the meaning "expected rate" and "observed rate". Information about the risk-adjustment procedure.

Erklärung: Erwartete und tatsächliche Rate

Erwartete Rate   Tatsächliche Rate

Bei diesem Qualitätsmerkmal wird für jedes Krankenhaus gesondert errechnet, bei wie viel Prozent der Patienten im Zusammenhang mit einer Ballonkatheterbehandlung der Tod zu erwarten ist. Grundlage für die Berechnung ist u.a. das so genannte Risiko-Profil der Patienten eines jeden Krankenhauses. Dieses Risiko-Profil hängt u.a. vom Alter, Geschlecht, von Vor- und Begleiterkrankungen der Patienten des Krankenhauses ab. Krankenhäuser mit vielen dieser Risiko-Patienten haben in der Regel eine höhere Rate an Todesfällen als Häuser mit wenig Risiko-Patienten. Die erwartete Rate wird anhand der anonymisierten Patientendaten des Vorjahres errechnet und dann mit der tatsächlichen Rate des darauf folgenden Jahres (Auswertungsjahr) verglichen. Ist die tatsächliche Rate gleich hoch wie die erwartete oder sogar geringer, zeugt das von guter Behandlungsqualität.

Erläuterungen zum Ergebnis

Die durchschnittliche tatsächliche Rate an verstorbenen Patienten im Zusammenhang mit einer Ballonkatheterbehandlung entsprach in Hamburg mit 2,69 Prozent exakt der erwarteten Durchschnittsrate. Die Hamburger Durchschnittsrate ist allerdings höher als die tatsächliche Rate auf Bundesebene (2,3 Prozent). Bei den Kliniken 4 und 5 ist die tatsächliche Rate an Todesfällen höher ausgefallen als die erwartete. Diese Ergebnisse sind auf gut begründete Einzelfälle zurückzuführen, die die medizinische Ergebnisqualität der betreffenden Krankenhäusern nicht beeinträchtigen.

Further explanations:
In Hamburg, the mean observed mortality rate is equal to the expected mean mortality rate (2.69 percent). However, the mean mortality rate in Hamburg is higher than the observed rate at the federal level (2.3 percent). For hospital 4 and 5, the observed mortality rate is higher than the expected rate. Those computational discrepancies could be plausibly explained. Thus, no qualitative discrepancies in the clinical area were concluded.

Portal E

Quality indicators → IQM-Qualitätsindikatoren

Hospitals	IQM-Qualitätsindikatoren	IQM-Zielwert Quelle (IQM Target value - Source)	IQM-Durchschnittswert Fallzahl 2012 (IQM Average - Number of patients treated)	Klinik-Ist-wert Fallzahl 2012 (Observed rate - Number of patients treated)	Klinik-Erwartungswert SMR 2012 (Expected rate - SMR)
Klinik 1	Todesfälle mit Linksherzkatheter bei Herzinfarkt<br>Alle Patienten > 19 Jahre	< Erwartungswert 4	5,4%<br>1.504 von 28.007	5,7%<br>18 von 315	8,4%<br>0,68
Klinik 2	Todesfälle mit Linksherzkatheter bei Herzinfarkt<br>Alle Patienten > 19 Jahre	< Erwartungswert 4	5,4%<br>1.504 von 28.007	5,3%<br>12 von 227	9,2%<br>0,58
Klinik 3	Todesfälle mit Linksherzkatheter bei Herzinfarkt<br>Alle Patienten > 19 Jahre	< Erwartungswert 4	5,4%<br>1.504 von 28.007	6,2%<br>24 von 390	7,9%<br>0,78
Klinik 4	Todesfälle mit Linksherzkatheter bei Herzinfarkt<br>Alle Patienten > 19 Jahre	< Erwartungswert 4	5,6%<br>1.384 von 24.661	6,5%<br>2 von 31	8,7%<br>0,74
Klinik 5	Todesfälle mit Linksherzkatheter bei Herzinfarkt<br>Alle Patienten > 19 Jahre	< Erwartungswert 4	5,4%<br>1.504 von 28.007	3,5%<br>22 von 622	8,0%<br>0,44

Everyday language description of the quality indicator

Portal F

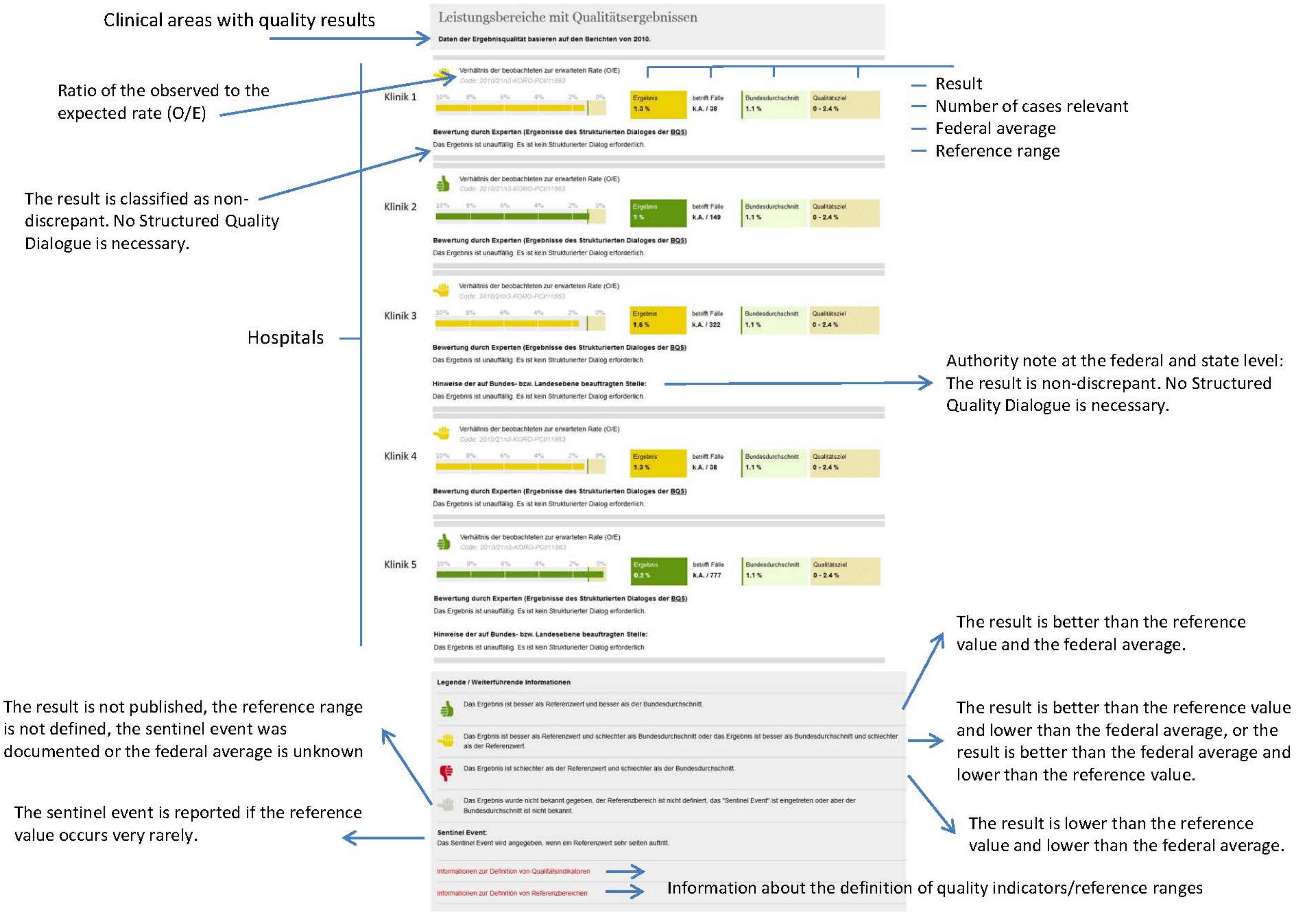
Clinical areas with quality results
Leistungsbereiche mit Qualitätsergebnissen
Daten der Ergebnisqualität basieren auf den Berichten von 2010.
Ratio of the observed to the expected rate (O/E)
Verhältnis der beobachteten zur erwarteten Rate (O/E)
Klinik 1
Ergebnis 1.3 %
betrifft Fälle k.A. / 38
Bundesdurchschnitt 1.1 %
Qualitätsziel 0 - 2.4 %
Result
Number of cases relevant
Federal average
Reference range
Bewertung durch Experten (Ergebnisse des Strukturierten Dialoges der BQS)
Das Ergebnis ist unauffällig. Es ist kein Strukturierter Dialog erforderlich.
The result is classified as non-discrepant. No Structured Quality Dialogue is necessary.
Klinik 2
Ergebnis 1 %
betrifft Fälle k.A. / 149
Klinik 3
Ergebnis 1.6 %
betrifft Fälle k.A. / 322
Hospitals
Hinweise der auf Bundes- bzw. Landesebene beauftragten Stelle:
Authority note at the federal and state level: The result is non-discrepant. No Structured Quality Dialogue is necessary.
Klinik 4
Ergebnis 1.3 %
betrifft Fälle k.A. / 38
Klinik 5
Ergebnis 0.2 %
betrifft Fälle k.A. / 777
Legende / Weiterführende Informationen
Das Ergebnis ist besser als Referenzwert und besser als der Bundesdurchschnitt.
The result is better than the reference value and the federal average.
Das Ergbnis ist besser als Referenzwert und schlechter als Bundesdurchschnitt oder das Ergebnis ist besser als Bundesdurchschnitt und schlechter als der Referenzwert.
The result is better than the reference value and lower than the federal average, or the result is better than the federal average and lower than the reference value.
Das Ergebnis ist schlechter als der Referenzwert und schlechter als der Bundesdurchschnitt.
The result is lower than the reference value and lower than the federal average.
Das Ergebnis wurde nicht bekannt gegeben, der Referenzbereich ist nicht definiert, das "Sentinel Event" ist eingetreten oder aber der Bundesdurchschnitt ist nicht bekannt.
The result is not published, the reference range is not defined, the sentinel event was documented or the federal average is unknown
Sentinel Event:
Das Sentinel Event wird angegeben, wenn ein Referenzwert sehr selten auftritt.
The sentinel event is reported if the reference value occurs very rarely.
Informationen zur Definition von Qualitätsindikatoren
Informationen zur Definition von Referenzbereichen
Information about the definition of quality indicators/reference ranges

Portal G

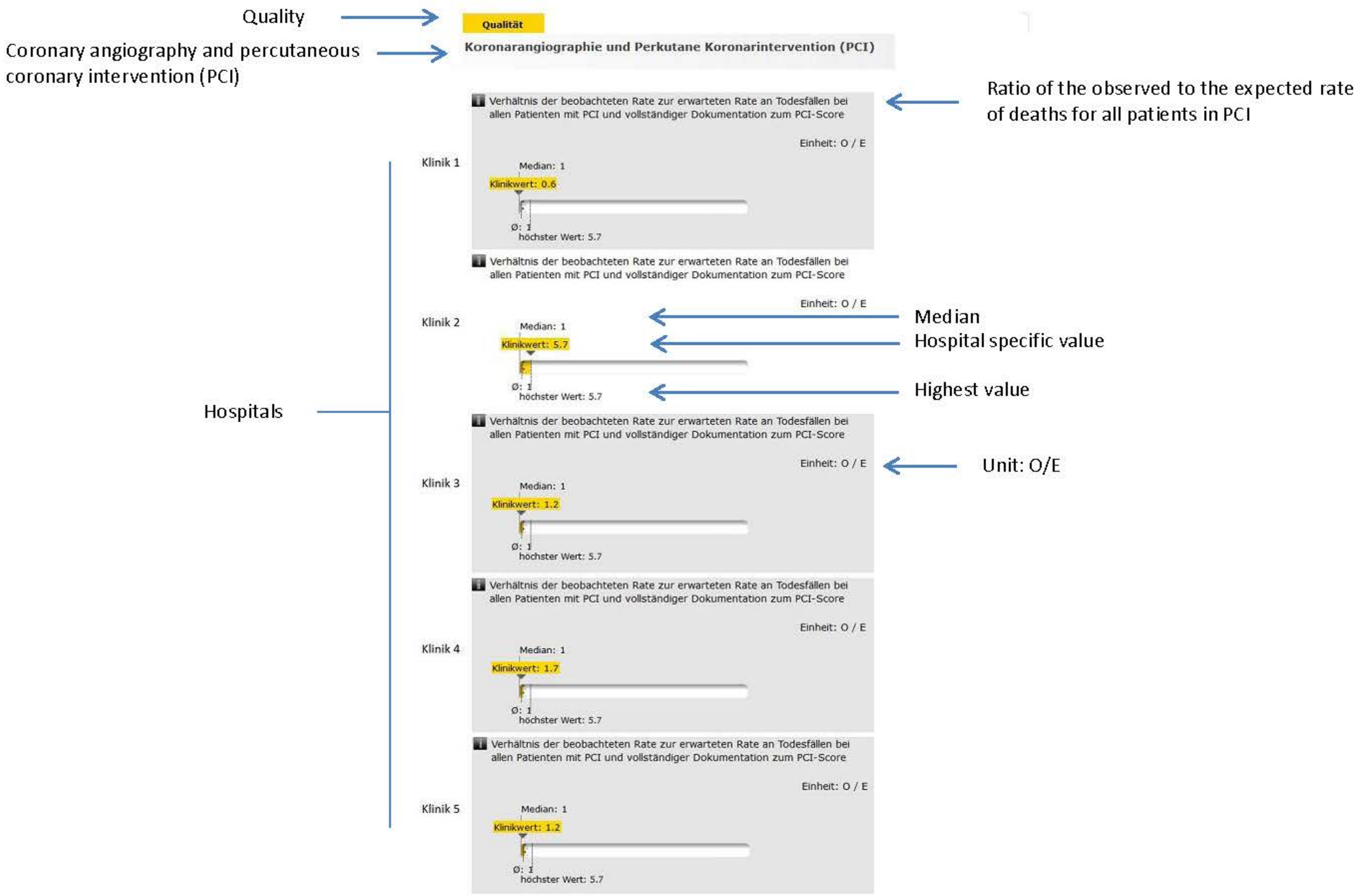

Quality
Qualität
Coronary angiography and percutaneous coronary intervention (PCI)
Koronarangiographie und Perkutane Koronarintervention (PCI)
Ratio of the observed to the expected rate of deaths for all patients in PCI
Hospitals
Median
Hospital specific value
Highest value
Unit: O/E
Klinik 1
Verhältnis der beobachteten Rate zur erwarteten Rate an Todesfällen bei allen Patienten mit PCI und vollständiger Dokumentation zum PCI-Score
Einheit: O / E
Median: 1
Klinikwert: 0.6
Ø: 1
höchster Wert: 5.7
Klinik 2
Verhältnis der beobachteten Rate zur erwarteten Rate an Todesfällen bei allen Patienten mit PCI und vollständiger Dokumentation zum PCI-Score
Einheit: O / E
Median: 1
Klinikwert: 5.7
Ø: 1
höchster Wert: 5.7
Klinik 3
Verhältnis der beobachteten Rate zur erwarteten Rate an Todesfällen bei allen Patienten mit PCI und vollständiger Dokumentation zum PCI-Score
Einheit: O / E
Median: 1
Klinikwert: 1.2
Ø: 1
höchster Wert: 5.7
Klinik 4
Verhältnis der beobachteten Rate zur erwarteten Rate an Todesfällen bei allen Patienten mit PCI und vollständiger Dokumentation zum PCI-Score
Einheit: O / E
Median: 1
Klinikwert: 1.7
Ø: 1
höchster Wert: 5.7
Klinik 5
Verhältnis der beobachteten Rate zur erwarteten Rate an Todesfällen bei allen Patienten mit PCI und vollständiger Dokumentation zum PCI-Score
Einheit: O / E
Median: 1
Klinikwert: 1.2
Ø: 1
höchster Wert: 5.7

Portal H

Here you can compare the quality of selected hospitals. A traffic lights system is used to illustrate the classification. The colors mean the following.

Green: non-discrepant
Yellow: reanalysis will be carried out
Red: discrepant

Ratio of the observed to the expected mortality rate by risk profiles

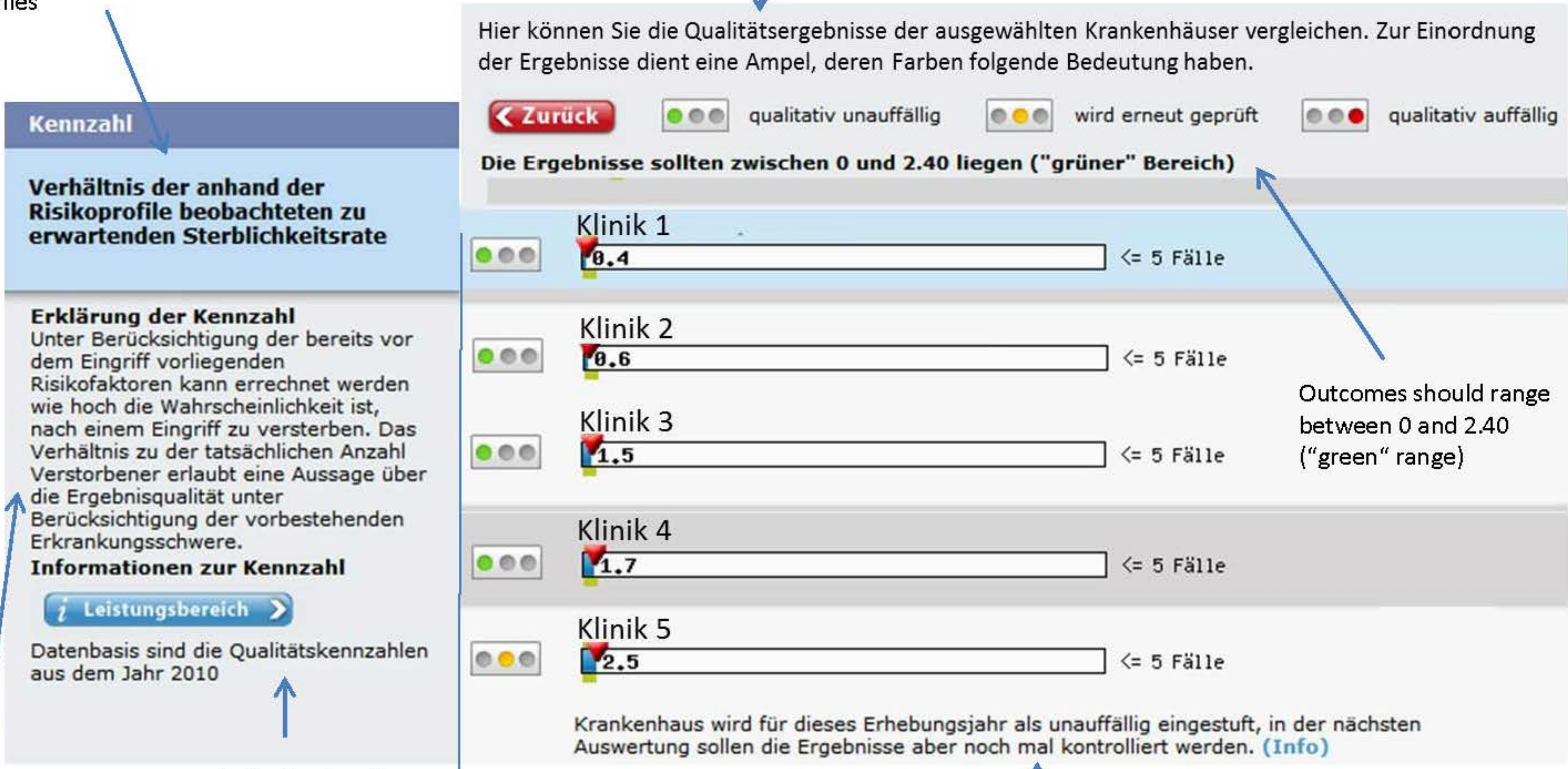

Outcomes should range between 0 and 2.40 (“green“ range)

Index Explanation

Hospitals

Explanation of the indicator:
Explanation of the meaning “expected rate” and “observed rate”. Information about the ratio of both rates.

Hospital is classified as non-discrepant this year; the results should be monitored again over the further analysis.

Portal I

Clinical areas: Coronary angiography and percutaneous coronary intervention

Leistungsbereich: Herz - Koronarangiographie und perkutane Koronarintervention

Auf diesen Seiten erhalten Sie Informationen über die Behandlungsergebnisse der Krankenhäuser. Erstmals sind die Kliniken verpflichtet,
einen Teil der von der Bundesgeschäftsstelle Qualitätssicherung (BQS) erhobenen Daten zu veröffentlichen.
Der Klinikführer stellt die Qualitätsindikatoren dar, die verpflichtend veröffentlicht werden müssen oder die zur Veröffentlichung empfohlen worden sind. Für etwa 1500 der rund 2000 Krankenhäuser liegen solche Daten zu medizinischen und pflegerischen Ergebnissen vor.

On these pages you receive information about treatment results of hospitals. For the first time, hospitals are obliged to publish a part of the collected data of the BQS. The Klinikführer presents quality indicators, which have to be published or which are recommended for publication. Such data is available for medical and nursing results for approximately 1,500 out of 2,000 hospitals.

Name of the quality indicator

Ratio of the observed to the expected rate (O/E)

Hospitals

	Name des Qualitätsindikators	Ergebnis	Referenzbereich	Bundesdurchschnitt	Bewertung
Klinik 1	Verhältnis der beobachteten zur erwarteten Rate (O / E)	0,7	<=2,4	1,1	●
Klinik 2	Verhältnis der beobachteten zur erwarteten Rate (O / E)	3,3	<=2,4	1,1	●
Klinik 3	Verhältnis der beobachteten zur erwarteten Rate (O / E)	0,1	<=2,4	1,1	●
Klinik 4	Verhältnis der beobachteten zur erwarteten Rate (O / E)	1,0	<=2,4	1,1	●
Klinik 5	Verhältnis der beobachteten zur erwarteten Rate (O / E)	1,7	<=2,4	1,1	●

● auffällig ● erneute Prüfung nötig ● unauffällig ● sonstiges

- Hospital result
- Reference range
- Federal average
- Evaluation

- Discrepant
- Reanalysis necessary
- Non-discrepant
- Other

Explanation of the Structured Quality Dialogue process

Weicht ein Krankenhaus vom vorgegebenen Referenzbereich ab, erhält es im "Strukturierten Dialog" Gelegenheit, einem Fachgremium diese Abweichung zu erläutern. Gelingt es ihm, zum Beispiel weil es besonders viele schwere Fälle behandelt hat, wird ihm dennoch gute Qualität attestiert. Gelingt es ihm nicht, wird es entweder im kommenden Jahr erneut geprüft oder als medizinisch auffällig eingestuft.

Der TK-Klinikführer macht diese Abstufung mit grünen, gelben und roten Markierungen sichtbar. Werden keine Daten abgebildet, so kann dies unter anderem daran liegen, dass der "Strukturierte Dialog" noch nicht abgeschlossen war, als das Krankenhaus seine Daten veröffentlicht hat. In diesen Fällen ist die Angabe von Daten zur Ergebnisqualität ausgeschlossen worden.

The TK-Klinikführer illustrates this classification in green, yellow and red. If there is no data presented, it can be possible (amongst others) that the Structured Quality Dialogue has not ended yet when the hospital has published its data. In this case, the reported data regarding the outcome is excluded.

Portal K

Hospital searching → **Krankenhaussuche**

Result lists → **Ergebnisliste**

Behandlungsanlass **Behandlung am Herzen bzw. den Herzkranzgefäßen mit Zugang über einen Schlauch (Katheter) entlang der Blutgefäße** [Anlass ändern]

Krankenhäuser 671-680 von 717: Gesetzliche Qualitätssicherung für "Untersuchung an den Herzkranzgefäßen bzw. Behandlung verengter Herzkranzgefäße mit einem eingeführten Schlauch (Herzkatheter)"

Häufigkeit von Todesfällen bei wiedereröffnender Behandlung von Herzkranzgefäßen unter Berücksichtigung des Risikoprofils von Patienten[1]

Die Qualitätskennzahl gibt die Antwort auf folgende Frage:

Wie häufig traten bei der wiedereröffnenden Behandlung von Herzkranzgefäßen Todesfälle auf (bestimmte Risikofaktoren wie das Alter der Patienten sind bereits berücksichtigt)?

← Short explantation about how to interpret the risk-adjusted quality indicator

Everyday language description of the risk-adjusted quality indicator → **Häufigkeit von Todesfällen bei wiedereröffnender Behandlung von Herzkranzgefäßen unter Berücksichtigung des Risikoprofils von Patienten**

Name	Ergebniswert	Bewertung durch den strukturierten Dialog	Kommentar / Erläuterung der zuständigen Stellen	Kommentar / Erläuterung des Krankenhauses
Klinik 1	4,40 %	●	-	-
Klinik 2	2,50 %	●	-	-
Klinik 3	0,30 %	●	-	-
Klinik 4	1,60 %	●	-	-
Klinik 5	3,60 %	●	-	-

Hospitals: Klinik 1–5

- Outcome
- Evaluation Structured Quality Dialogue
- Comment competent authority
- Comment hospital

Legende:

- ● Ergebnis qualitativ unauffällig → Outcome non-discrepant
- ● Ergebnis qualitativ auffällig → Outcome discrepant
- ● Ergebniseinstufung noch nicht abgeschlossen → Assessment incomplete
- K Sonstiges (im Kommentarfeld erläutert) → Other
- \- Es liegen keine Daten vor. → No data available

1) **Weiterführende Informationen zur Qualitätskennzahl:**

Bundesweiter Referenzbereich für diese Qualitätskennzahl: 0 bis höchstens 5,8 Prozent

Die Zuverlässigkeit und Unterscheidungsfähigkeit der Qualitätskennzahl wird als gut beschrieben.

Die Qualitätskennzahl wurde in der abschließenden Bewertung der AQUA-Fachkommission als verpflichtend zur Veröffentlichung in den Strukurierten Qualitätsberichten der Krankenhäuser empfohlen.

Kommentar der Fachkommission:
Gute Bewertung (Expertenbefragung). Empirie gut.

← Further information about the quality indicator including the federal referance range, the suitability for publiation, and comments of the Federal Joint Committee
